# Salicylate of Soda in Rheumatism

**Published:** 1879-08

**Authors:** 


### SALICYLATE OF SODA IN RHEUMATISM.

Salicylate of soda need rarely be given in larger doses than from one to three drams daily in order to produce all its best effects, which will be marked in one or two, or three days at most: and then the dose should be gradually diminished until some time after complete convalescence has set in.—*Maryland Medical Journal.*

